# Patient participation in forensic psychiatric care: Mental health professionals' perspective

**DOI:** 10.1111/inm.12806

**Published:** 2020-10-23

**Authors:** Mikael Selvin, Kjerstin Almqvist, Lars Kjellin, Agneta Schröder

**Affiliations:** ^1^ University Health Care Research Center Faculty of Medicine and Health Örebro University Örebro Sweden; ^2^ Department for Social and Psychological Studies Karlstad University Karlstad Sweden; ^3^ Department of Health Science Faculty of Health, Care and Nursing Norwegian University of Science and Technology (NTNU) Gjövik Norway

**Keywords:** forensic psychiatry, inpatients, patient participation, psychiatric nursing, quality of health care

## Abstract

Patient participation is a central concept in modern health care and an important factor in theories/models such as person‐centred care, shared decision‐making, human rights approaches, and recovery‐oriented practice. Forensic psychiatric care involves the treatment of patients with serious mental illnesses who also have committed a crime, and there are known challenges for mental health staff to create a health‐promoting climate. The aim of the present study was to describe mental health professionals' perceptions of the concept of patient participation in forensic psychiatric care. Interviews were conducted with 19 professionals and were analysed with a phenomenographic approach. The findings are presented as three descriptive categories comprising five conceptions in an hierarchic order: 1. create prerequisites – to have good communication and to involve the patient, 2. adapt to forensic psychiatric care conditions – to take professional responsibility and to assess the patient’s current ability, and 3. progress – to encourage the patient to become more independent. The findings highlight the need for professionals to create prerequisites for patient participation through good communication and involving the patient, whilst adapting to forensic psychiatric care conditions by taking professional responsibility, assessing the patient’s ability, and encouraging the patient to become more independent without adding any risks to the care process. By creating such prerequisites adapted to the forensic psychiatric care, it is more likely that the patients will participate in their care and take more own responsibility for it, which also may be helpful in the patient recovery process.

## Introduction

Patient participation is a central concept in modern health care and an important factor in theories/models such as person‐centred care (Ekman *et al*. 2011), shared decision‐making (Patel *et al*. 2008), human rights approaches (Gruskin *et al*. 2007), and recovery‐oriented practice (Chester *et al*. 2016). In recent years, the patients’ position has been strengthened and positive effects of involving the patient in their care have been highlighted in research, for example, by increased safety (Weingart *et al*. 2011) and patient satisfaction (Dwamena *et al*. 2012). This means that healthcare professionals not only have a responsibility to encourage patient participation, but it also seems to be an important aspect in delivering high‐quality care.

## Background

The concept of patient participation is complex, and definitions of it vary depending on the context and perspective (Eldh *et al*. 2006). Complex factors such as staff and patients’ attitudes, competence, environment, motivation, and health status seem to directly or indirectly affect patient participation (Sahlsten *et al*. 2008; Tobiano *et al*. 2015b). In previous studies, researchers tried to clarify the concept of patient participation from a nursing perspective. Sahlsten *et al*. (2007) emphasize the dynamic nurse–patient interaction process and describe the concept from a core category called *Mutuality in negotiation. Mutuality* includes exchange and co‐operation between nurse and patient, and the nurse should, based on professionalism, balance the patient’s unique abilities with actual needs. *Negotiation* means a dynamic interaction and the nurse is supposed to supply knowledge adapted to the patient, a prerequisite for making informed choices. Tobiano *et al*. (2015a) state that the nurse’s role in enacting participation should always build on respect of the patient’s knowledge, honour of their choices, and the professional responsibility to enable participation by encouragement and providing information to the patient. There is also, however, the need for accommodating and assessing each patient’s risks and characteristics and to regulate information and medication when needed. This requires that the nurse is aware of attitudes, status, and cultural aspects among both patients and staff that could hinder participation.

Forensic psychiatric care involves the treatment of patients with serious mental illnesses who have also committed a crime (Nedopil *et al*. 2015). This setting can present a challenge for mental health staff for creating a health‐promoting climate (Hörberg 2008). The care is coercive in the meaning that the patients are involuntarily admitted, and compared with other forms of psychiatric care, the patients also tend to show more multiple disabilities, including antisocial behaviour, substance misuse, and poor insight and adherence to treatment (Gordon & Lindqvist 2007). These complex circumstances could explain two interesting findings from previous studies of quality of forensic psychiatric care; the patients often experience that the quality of the care is lower than the staff do, and both patients and staff rate items regarding patient participation as low (Lundqvist *et al*. 2014; Lundqvist & Schröder 2015; Schröder *et al*. 2013; Selvin *et al*. 2019). This is of interest because according to the law, the patient should be active and participate in their care even if the patient autonomy is limited (Swedish Forensic Mental Care Act; 1991:1129).

Previous research about patient participation in forensic psychiatric care highlights diverse understandings and abilities in an inflexible setting that patients have different abilities to participate and the secure setting itself is a hindering factor for nurses to promote patient participation (Magnusson *et al*. 2019). Söderberg *et al*. (2019) emphasize the difficulty of balancing the paradoxical role of caring for the patient’s interests and development, whilst simultaneously representing and adhering to the rules and regulations in the system in which one is employed. However, based on the complexity of both the concept and the setting itself, we believe that knowledge about the concept in this context is missing. In a previous interview study, patients in forensic psychiatric care were asked how they perceive the concept (Selvin *et al*. 2016), but we also need to add more perspectives in order to achieve a broader understanding of the nature of participation. Therefore, the aim of the present study was to describe mental health professionals' perceptions of the concept of patient participation in forensic psychiatric care.

## Methods

### Setting

The study group consisted of mental health professionals working at two different forensic psychiatric clinics in Sweden. Both clinics had a medium security classification with patients admitted according to the Swedish Forensic Mental Care Act (1991:1129). This refers to patients who had committed a crime which would normally have led to prison, but because of serious mental illnesses, they were transferred to forensic psychiatric care. In Sweden, this care is not time‐specific, but before discharge the patient must have a permanent home, structured days with regular activities and there must be a low risk of committing new crimes. The two clinics were similar in size, each with around 25‐bed sites and a staff of around 100 employees.

### Participants

In total, 19 professionals participated in the study (ten women and nine men) comprising five nursing assistants, seven nurses, two counsellors, two psychologists, and three psychiatrists. The average age was 48 years (range: 26–68), and their working experience within forensic psychiatric care ranged from one year to 30 years.

### Recruitment and data collection

Data were collected through individual interviews. The selection of healthcare professionals for interviews was strategic, and the purpose was to get as broad variation as possible regarding gender, profession, and experience. We had contact persons at the clinics to help with the logistics of data collection. Those selected for participation were informed orally and in writing about the aim of the study, their right to withdraw from the study at any moment and that their participation was voluntary. The researchers were available if any questions were forthcoming. All of the professionals who were asked to participate were interested, and they were contacted by the first author by telephone, e‐mail, or personal meeting, to schedule interviews. All participants gave written consent to participate before the interview started, and the study was approved by the regional research ethics committee in Uppsala (reference number 2017/144).

The first author, a registered psychiatric nurse with a clinical background in forensic psychiatric care, conducted all the interviews. The interviews began with the same main question: 'How do you perceive the concept of patient participation in forensic psychiatric care?' Open‐ended questions were asked occasionally, depending on how fully the person answered the main question. For example: 'What do you mean?', 'Can you develop that more?' or 'With your experience, can you describe a situation …?' All interviews were carried out at the clinics in quiet rooms separated from the wards, between June and August 2017. The length of the interviews was 30–60 min, and they were recorded and transcribed verbatim. In total, there were 302 pages of transcribed text.

### Data analysis

The data were analysed using a phenomenographic approach, and this was chosen to describe the variation in how the informants perceived the concept of patient participation. This method has a pedagogical outset and makes a distinction between two perspectives in describing knowledge: the first‐order perspective, what something is, and the second‐order perspective, how something is perceived to be (Marton 1981; Marton & Booth 1997). In phenomenographic studies, the aim is to identify the second‐order perspective and to describe the various ways in which people experience, understand, or conceive certain phenomena in the world around them (Marton 1981, 1992, 1994). The findings are illustrated in different categories, and the researcher needs to show how those are related to each other. Normally, a hierarchy is created where a progression from the fundamental ways of understanding the phenomenon to more complex and developed conceptions are described. The result is a description, on a collective level, of the logical relationship between conceptions and the descriptive categories (Marton 1986, 1994; Marton & Booth 1997).

The analysis was carried out in four steps and was not linear because the researcher must go back and forth during the process (Marton 1986). Firstly, the whole text was read with an open mind until the data were familiar, and then with further reading, 227 statements relevant to the aim were identified. In the second step, we identified similarities and differences between the informants’ descriptions of the phenomenon and labelled those into preliminary conceptions. The third step consisted of comparing and grouping the conceptions into preliminary descriptive categories in order to capture the meaning of the concept of patient participation. During the fourth step, three descriptive categories that describe the variation of the concept of patient participation emerged.

## Findings

**Fig. 1:**
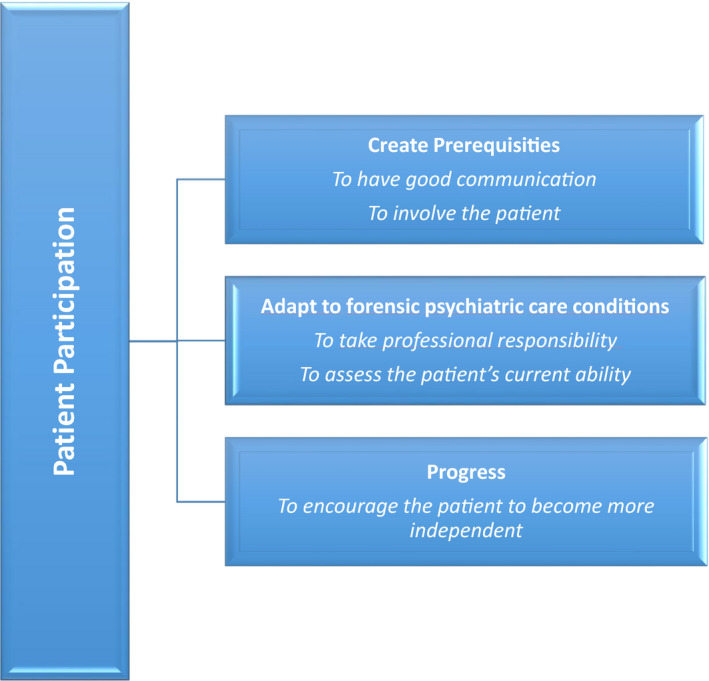
Patient participation in forensic psychiatric care: mental health professionals' perspective.

The findings are presented as three descriptive categories comprising five conceptions in a hierarchic order (Fig. 1): 1. create prerequisites – to have good communication and to involve the patient, 2. adapt to forensic psychiatric care conditions – to take professional responsibility and to assess the patient’s current ability, and 3. progress – to encourage the patient to be become more independent. The conceptions are illustrated with quotations from interviews.

## Create prerequisites

This descriptive category is composed of two conceptions that relate to good communication and involvement of the patient, which, according to the professionals in this study, are prerequisites for promoting patient participation. The informants described that patient participation demanded mutual engagement through active communication and that staff should encourage the patient to be active and involved. It was not only a question about the patients' ability or motivation, but it was also crucial from a professional perspective.

### To have good communication

The informants described that it was important that the patient got sufficient information in a way that was clear and easy to understand. Depending on the situation, they adjusted their way of communicating in relation to timing, method of communication, and the patient’s ability to understand. A lot of different types of information need to be processed, of both positive and sometimes negative meaning for the patient. This can be anything from daily talk, to more complex discussions such as care planning, discharge, and treatment options. In this form of care, the patients are in a vulnerable position and the professionals emphasized their own responsibility for 'guiding' the patient.

I talk to them, and if they receive written information I explain and help them understand if necessary. I also tell them what is planned, sometimes what has happened and why things happen. (Interview 8)

The informants also described the importance of having good communication within the professional group, for example, by keeping good case reports and by communicating relevant issues to colleagues. By doing this, they felt that it was easier to work in a united manner and to act professionally with the patients. However, they also described hindrances in communication, such as long and tedious processes in relation to receiving information and coming to decisions regarding the patient.

We write in the journal what we have done with the patient and what we have talked about. It is important that this information is accessible, no matter who is working at the moment, so the work with the patient can continue. Sometimes the patients forget what has been said and what we have agreed. (Interview 19)

### To involve the patient

The informants described the importance of giving the patient the opportunity to be involved and also for being aware that the patients are in a dependent situation. The professionals perceived that with patients with low insight and motivation, they often needed to encourage the patient to be involved, and to listen and act according to the patients’ wishes. The informants also described that they often saw that vulnerable patients were thankful when they had a sense of involvement.

It could be anything from being asked in the daily life about “small things”, to being a part in the decision making as long as possible. And that the patient is involved in the care process, for example by planning the care. (Interview 1)

Sometimes it is necessary to have meetings without the patient. In such cases, the informants still experienced that the patient could be involved with some beforehand planning and then through discussing matters with the patient afterwards. Also, in situations where decisions are made against the patient’s will, the informants still experienced that is important to involve the patient and to explain why it was so. The professionals described that full participation in forensic psychiatric care cannot be achieved, but they can always involve the patient in the best way possible.

Maybe the patients can’t choose if they should have medication or not, but I can give them the opportunity to choose between similar medications, and also ask them what they have tried before, which effect it had and what they want now. (Interview 18)

## Adapt to forensic psychiatric care conditions

This descriptive category is composed of two conceptions, *to take professional responsibility* and *to assess the patient's current ability*, and it relates to professionalism, responsibility, and the fact that patients in forensic psychiatric care often have serious mental illnesses which could affect their ability to participate.

### To take professional responsibility

The professionals described situations where they, based on their professional experience and knowledge, sometimes had the responsibility to adjust the patient's own planning and sometimes to even go against the patient's will. According to the professionals, the patient should be involved to a greater or lesser extent, depending on the situation. Apart from the patient’s own requests, the professionals described that they must also have regard for aspects of the law, their responsibility to the society, and the risk for criminality.

In my profession, saying no is a part of the job, that is included, but in the same way it is also my responsibility during certain circumstances. (Interview 7)

They are here for a reason. We can’t demand that they can take care of themselves. If the patients could manage and take all right decisions, they shouldn’t be here (Interview 11)

Participation in the care does not mean that the patient should decide everything. Sometimes the right thing is to make decisions against the patient’s will even if it is a challenge for the professionals to handle this in a balanced way. They also described this as being an important part of the job. The informants perceived that it is common that patients are not always fully aware of their problems and how to work with them in an effective way, and therefore, it is necessary to have professional responsibility.

If the patient has low insight, is seriously ill with ongoing psychosis for example and perhaps is even dangerous, it obviously affects the possibility for participating in the care. In that case the patient cannot be active in all parts of the process. For me, participation correlates with insight. (Interview 2)

### To assess the patient’s current ability

The informants said that they sometimes have to deal with all kinds of situations, everything from active patients with a lot of ambitious, but unrealistic goals, to patients who are really hard to motivate. These differences and variations could also be true with the same patient.

One day the patient is highly motivated and everything sounds fantastic. The next day it is like he has forgotten what we talked about. (Interview 15)

The professionals perceived that it is important to adjust their approach depending on the situation; it is not possible to treat every patient in the same way. On a ward with many patients, there are also individual variations and the informants emphasized that as professionals they must be aware and distribute their time according to the patients' needs – not only in relation to how active the patients are.

I have a few patients that I am more responsible for, I am their contact person on the ward and it is clear that I have to adjust my way of acting depending on each individual patient. Some patients need a lot of pushing and some patients are quite independent and they come to me when they want something. (Interview 6)

## Progress

This descriptive category is composed of one conception, *to encourage the patient to be more independent,* and it relates to the patient’s own responsibility. The informants described that patient participation can be a tool for more effective treatment because it is one way to work with patients' insight and motivation in a positive way. The goal with forensic psychiatric care is that the patients should be able to improve their own mental health and their functional level, which could eventually lead to discharge, even though this may be a long process.

### To encourage the patient to become more independent

The professionals perceived that patient participation is a process of gradually increasing involvement and of being encouraging over time, which often leads to improved self‐confidence and awareness with the patients. The patients also became more motivated, and the professionals experienced that participation could be used towards planning discharge. When planning included the patient, the professionals noticed a higher degree of adherence. They also found it valuable to work constantly with the patients' own responsibility‐taking by giving feedback regarding outcome compared with planning.

OK, you just had your leave. It worked, you came back when you should and no alcohol or drugs. That’s a big step forward! Next time we can expand the time. (Interview 12)

Small steps at a time. How I explain constantly and how I encounter the patient is important. After a while, the patients often change their mind and see that it works. (Interview 17)

## Discussion

In this study, we sought to examine health professionals' perceptions of the concept of patient participation in forensic psychiatric care. We expected to find similarities to other studies, but also important differences according to the specific context. Our findings support previous research that the concept of patient participation is complex (Eldh *et al*. 2006; Sahlsten *et al*. 2007; Tobiano *et al*. 2015a).

It seems to be important to create prerequisites for participation by having good communication and involving the patient. This is similar to findings in previous research (Eldh 2006; Sahlsten *et al*. 2008; Tobiano *et al*. 2015b). What is noteworthy though is how the professionals in this study described their own responsibility in the process. They emphasized the need to see each individual patient and to act according to the situations and that professionals should adjust their approach based on the patient's function, actual situation, and the fact that there is a power imbalance. This relates to building an alliance between clinician and patient, which can be achieved by collaborative partnerships, personal bonds, and mutual agreement (Chakrabarti 2018). In forensic psychiatric care, however, the professionals balance coercive and law‐related aspects with creating and maintaining the carer–patient relationship. Söderberg *et al*. (2019) describe this as *a good relationship: not participation but the next best thing*. Full participation can perhaps not be achieved, but by building good relations with patients through trust, security, and communication, conditions for supporting patient participation are created. The carer–patient relationship is also something that Magnusson et al. highlight (2019) as important in promoting patient participation in forensic psychiatric care. We can assume that the chances of achieving alliance and trust, which is important for patient participation, increase with good communication and involvement of the patient.

An interesting finding was that professionals in forensic psychiatric care feel that they sometimes have to take professional responsibility and act in a different manner to how the patient would like. They seem to reason that this is a part of their job because of characteristics of the patients, such as low insight, lack of ability, and also from a legal perspective. From a patient perspective, we know that staff can assess the situation and capabilities differently than the patients (Selvin *et al*. 2019; Selvin *et al*. 2016). We also know that patients describe experiences of frustration due to their not being listened to and the fact that they had to adapt to the ward, the staff, and rules, and this attenuated their ability to improve their health (Marklund *et al*. 2019). Differences in patients' and professionals' experiences are perhaps expected, but it is important to reflect upon why these differences exist and also to try to prevent them as much as possible (Selvin *et al*. 2019). Differences could for example exist as a result of involuntarily actions, discipline, and power abuse (Hörberg & Dahlberg 2015). Based on our results, it is necessary in certain circumstances to go against the patient’s will, but the outcome could differ depending on how the professionals act. We suggest that professionals should involve the patient when possible and for example explain why decisions are made, treat the patient with respect, and evaluate and give regular feedback to the patient during the care process.

According to the health professionals in our study, patient participation can be a means for encouraging the patient to become more independent. It is important though, as a staff member, to support the patient in the process and assess every individual situation. Patients in forensic psychiatric care experience positive progression in their treatment when they are active and take the initiative, but they describe how important it is that staff help them to take the initiative during periods of reduced mental health (Selvin *et al*. 2016). This is similar to what Sahlsten (2008) termed *mutual engagement*. Patients also perceive that participation in forensic psychiatric care is more than decisions about medicines and adherence to predetermined treatment; it is also about being able to participate in determining their own future (Marklund *et al*. 2019). Based on our findings, we can assume that professionals in forensic psychiatric care do try to achieve more active patient participation, but there are also hindering factors to be aware of, for example, the challenge of knowing when to start allowing patients to participate in care and to what degree (Schandl *et al*. 2017). A recent qualitative meta‐synthesis of participation in mental health care emphasized the concept of influence, and it was also found that staff take more responsibility during periods when the patient is seriously ill (Stomski & Morrison 2017). However, it could become a dilemma for professionals to try to keep a balance and to know when to take more or less responsibility, especially in coercive care (Sjöström 2006).

A dilemma of being a caregiver in forensic psychiatric care is a situation where ethical norms, community protection, autonomy, and justice versus the patient’s well‐being make it hard for professionals to always act in line with the patient’s own will (Ward 2013). Working in this climate can be challenging, and it is common that healthcare personnel have problems with emotional exhaustion (López‐López *et al*. 2019). It seems that nurses in forensic psychiatric care experience difficulties and that they express diverse understandings and abilities in an inflexible setting, and that participation is a word used in research but difficult to embrace in clinical practice because patients do not understand and interpret the concept in the same way as staff do (Magnusson *et al*. 2019). Our study has contributed more knowledge about the concept of patient participation in forensic psychiatric care. Further research should investigate the association between patient participation and quality of forensic psychiatric care.

## Strengths and limitations

Forensic psychiatric care is based on teamwork, and the treatment is so complex that different competencies are necessary to achieve success. Therefore, a strength in this study was the variety of mental health professionals and the representation of two different clinics. We also found that the chosen method was effective for the aim of this study. A limitation may be the authors’ preunderstanding. The first author has experience from working as a forensic nurse and from previous research regarding patient participation. Even if all authors were involved and discussed the findings until consensus, it is possible that interviews and the process of analysis were affected and the results should be interpreted accordingly. We acknowledge that the research was conducted in one country and the services available may not match other jurisdictions.

## Relevance for clinical practice

The findings in this study contribute important knowledge that can encourage reflection and accordingly help mental health professionals and decision makers to create an environment that promotes patient participation in forensic psychiatric care. It is important though to be aware that these results are a reflection of the healthcare professionals’ perceptions which is just one type of perspective. Therefore, other perspectives – and especially the patient perspective – should also be considered in the planning and implementation of interventions for improving patient participation.

## Conclusion

Patient participation seems to be important in order to provide high‐quality care. Based on our findings, however, we confirm some of the challenges of patient participation in coercive care that has been highlighted in previous research. Our findings highlight the need for professionals to create prerequisites for patient participation through good communication and involving the patient, whilst adapting to forensic psychiatric care conditions by taking professional responsibility, assessing the patient’s ability, and encouraging the patient to become more independent without adding any risks to the care process. By creating such prerequisites adapted to the forensic psychiatric care, it is more likely that the patients will participate in their care and take more own responsibility for it, which also may be helpful in the patient recovery process.

## Funding

No funding was received for this study.

## References

[inm12806-bib-0001] Chakrabarti, S. (2018). Treatment alliance and adherence in bipolar disorder. World Journal of Psychiatry, 8 (5), 114–124. 30425942 10.5498/wjp.v8.i5.114 PMC6230924

[inm12806-bib-0002] Chester, P. , Ehrlich, C. , Warburton, L. , Baker, D. , Kendall, E. & Cromton, D. (2016). What is the work of Recovery Oriented Practise? A Systematic literature review. International Journal of Mental Health Nursing, 25, 270–285. 27381002 10.1111/inm.12241

[inm12806-bib-0003] Dwamena, F. , Holmes‐Rovner, M , Gaulden, C. M. *et al*. (2012). Interventions for providers to promote a patient‐centred approach in clinical consultations. The Cochrane database of Systematic Reviews, 12, CD003267. 23235595 10.1002/14651858.CD003267.pub2 PMC9947219

[inm12806-bib-0004] Ekman, I. , Swedberg, K , Taft, C *et al*. (2011). Person‐centered care – Ready for prime time. European Journal of Cardiovascular Nursing, 10, 248–251. 21764386 10.1016/j.ejcnurse.2011.06.008

[inm12806-bib-0005] Eldh, A. C. (2006). Patient participation: what it is and what it is not. Diss. Department of Caring Sciences, Örebro University http://www.diva‐portal.org/smash/get/diva2:137201/FULLTEXT02.pdf.

[inm12806-bib-0006] Eldh, A. C. , Ehnfors, M. & Ekman, I. (2006). The meaning of patient participation for patients and nurses at a nurse‐led clinic for chronic heart failure. European Journal of Cardiovascular Nursing, 5, 45–53. 16014340 10.1016/j.ejcnurse.2005.06.002

[inm12806-bib-0007] Gordon, H. & Lindqvist, P. (2007). (2007) Forensic psychiatry in Europe. Psychiatric Bullentin, 31 (11), 421–424.

[inm12806-bib-0008] Gruskin, S. , Mills, E.‐J. & Tarantola, D. (2007). History, principles, and practice of health and human rights. Lancet, 370, 449–455. 17679022 10.1016/S0140-6736(07)61200-8

[inm12806-bib-0009] Hörberg, U. (2008). To be the subject of care or the object of correction. Forensic psychiatric care and the power of tradition. Diss. Faculty of Health Science and Social Work, Växjö University http://www.divaportal.org/smash/get/diva2:205815/FULLTEXT01.pdf.

[inm12806-bib-0010] Hörberg, U. & Dahlberg, K. (2015). Caring potentials in the shadows of power, correction and discipline – Forensic psychiatric care in the light of the work of Michel Foucault. International Journal of Qualitative Studies on Health and Well‐being, 10 (1), 28703–28709. 26319100 10.3402/qhw.v10.28703 PMC4552868

[inm12806-bib-0011] López‐López, I. M. , Gómez‐Urquiza, J. L. , Cañadas, G. R. , De la Fuente, E. I. , Albendin‐Garcia, L. & Cañadas‐De la Fuente, G. A. (2019). Prevalence of burnout in mental health nurses and related factors: a systematic review and meta‐analysis. International Journal of Mental Health Nursing, 28, 1035–1044. 10.1111/inm.12606 31132216

[inm12806-bib-0012] Lundqvist, L. O. , Lorentzen, K. , Riiskjaer, E. & Schröder, A. (2014). A Danish adaptation of the Quality in Psychiatric Care – Forensic In‐Patient questionnaire: Psychometric properties and factor structure. Journal of Forensic Nursing, 10 (3), 168–174. 25144588 10.1097/JFN.0000000000000036

[inm12806-bib-0013] Lundqvist, L. O. & Schröder, A. (2015). Patient and staff views of quality in forensic psychiatric inpatient care. Journal of Forensic Nursing, 11 (1), 51–58. 25695210 10.1097/JFN.0000000000000060

[inm12806-bib-0014] Magnusson, E. , Axelsson, A. K. & Lindroth, M. (2019). ´We try´‐ how nurses work with patient participation in forensic psychiatric care. Scandinavian Journal of Caring Sciences, 34 (3), 690–697. 31749183 10.1111/scs.12773

[inm12806-bib-0015] Marklund, L. , Wahlroos, T. , Ejneborn, G.‐M. & Gabrielsson, S. (2019). ‘I know what I need to recover’: Patients´ experiences and perceptions of forensic psychiatric care. International Journal of Mental Health Nursing, 29 (2), 235–243. 31642598 10.1111/inm.12667

[inm12806-bib-0016] Marton, F. (1981). Phenomenography—Describing conceptions of the world around us. International Science, 10, 177–200.

[inm12806-bib-0017] Marton, F. (1986). Phenomenography—A research approach to investigating different understanding of reality. Journal of Thought, 21, 28–49.

[inm12806-bib-0018] Marton, F. (1992). Phenomenography and “The art of teaching all things to all men”. International Journal of Qualitative Studies in Education, 5, 253–267.

[inm12806-bib-0019] Marton, F. (1994). Phenomenography. In: T. Husén & T. N. Postlethwaite (Eds). International Encyclopedia of Education, 2nd edn., vol 8 (pp. 4424–4429). Oxford; Pergamon.

[inm12806-bib-0020] Marton, F. & Booth, S. (1997). Learning and Awareness. Mahwah, NJ: Lawrence Erlbaum Associates.

[inm12806-bib-0021] Nedopil, N. , Taylor, P. & Gunn, J. (2015). Forensic psychiatry in Europe: The perspective of the Ghent group. International Journal of Psychiatry in Clinical Practice, 19, 80–83. 25263225 10.3109/13651501.2014.967700

[inm12806-bib-0022] Patel, S.‐R. , Bakken, S. & Ruland, C. (2008). Recent advances in shared decision making for mental health. Current Opinions of Psychiatry, 21 (6), 606–612. 10.1097/YCO.0b013e32830eb6b4 PMC2676935 18852569

[inm12806-bib-0023] Sahlsten, M. J. M. , Larsson, I. E. , Sjöström, B. , Lindencrona, C. S. C. & Plos, K. A. E. (2007). Patient participation in nursing care: towards a concept clarification from a nurse perspective. Issues in Clinical Nursing, 16 (4), 630–637. 10.1111/j.1365-2702.2006.01660.x 17402943

[inm12806-bib-0024] Sahlsten, M. J. M. , Larsson, I. E. , Sjöström, B. & Plos, K. A. E. (2008). An analysis of the concept of patient participation. Nursing Forum, 43 (1), 2–11. 18269439 10.1111/j.1744-6198.2008.00090.x

[inm12806-bib-0025] Schandl, A. , Falk, A.‐C. & Frank, C. (2017). Patient participation in the intensive care unit. Intensive and Critical Care Nursing, 42, 105–109. 28539205 10.1016/j.iccn.2017.04.006

[inm12806-bib-0026] Schröder, A. , Ågrim, J. & Lundqvist, L.‐O. (2013). The quality in psychiatric care – forensic in‐patient instrument: psychometric properties and patient views of the quality of forensic psychiatric services in Sweden. Journal of Forensic Nursing, 9 (4), 225–234. 24256985 10.1097/JFN.0b013e31827f5d2f

[inm12806-bib-0027] Selvin, M. , Almqvist, K. , Kjellin, L. , Lundqvist, L.‐O. & Schröder, A. (2019). Patient and staff experiences of quality in Swedish forensic psychiatric care: a repeated cross‐sectional survey with yearly sampling at two clinics. International Journal of Mental Health Systems, 13, 8. 30733827 10.1186/s13033-019-0265-z PMC6359846

[inm12806-bib-0028] Selvin, M. , Almqvist, K. , Kjellin, L. & Schröder, A. (2016). The concept of patient participation in forensic psychiatric care: the patient perspective. Journal of Forensic Nursing, 12 (2), 57–63. 27088759 10.1097/JFN.0000000000000107

[inm12806-bib-0029] Sjöström, S. (2006). Invocation of coercion context in compliance communication – power dynamics in psychiatric care. International Journal of Law and Psychiatry, 29 (1), 36–47. 16309742 10.1016/j.ijlp.2005.06.001

[inm12806-bib-0030] Söderberg, A. , Wallinius, M. & Hörberg, U. (2019). An interview study of professional carers´ experiences of supporting patient participation in a maximum security forensic psychiatric setting. Issues in Mental Health Nursing, 41 (3), 201–210. 31765239 10.1080/01612840.2019.1658833

[inm12806-bib-0031] Stomski, N. J. & Morrison, P. (2017). Participation in mental healthcare: a qualitative meta‐synthesis. International Journal of Mental Health Systems, 11, 67. 29151851 10.1186/s13033-017-0174-y PMC5678577

[inm12806-bib-0032] Tobiano, G. , Bucknall, T. , Marshall, A. , Guinane, J. & Chaboyer, W. (2015a). Nurses’ views of patient participation in nursing care. Journal of Advanced Nursing, 71 (12), 2741–2752. 26216742 10.1111/jan.12740

[inm12806-bib-0033] Tobiano, G. , Marshall, A. , Tracey, B. & Chaboyer, W. (2015b). Patient participation in nursing care on medical wards: An integrative review. International journal of Nursing Studies, 52, 1107–1120. 25769475 10.1016/j.ijnurstu.2015.02.010

[inm12806-bib-0034] Ward, T. (2013). Addressing the dual relationship problem in forensic and correctional practice. Aggression and Violent Behavior, 18 (1), 92–100.

[inm12806-bib-0035] Weingart, S. N. , Zhu, J. , Chiappetta, L. *et al*. (2011). Hospitalized patients’ participation and its impact on quality of care and patient safety. International Journal for Quality in Health Care, 23 (3), 269–277. 21307118 10.1093/intqhc/mzr002 PMC3140261

